# Current Aspects on the Pathophysiology of Bone Metabolic Defects during Progression of Scoliosis in Neurofibromatosis Type 1

**DOI:** 10.3390/jcm11020444

**Published:** 2022-01-15

**Authors:** Angelos Kaspiris, Olga D. Savvidou, Elias S. Vasiliadis, Argyris C. Hadjimichael, Dimitra Melissaridou, Stella Iliopoulou-Kosmadaki, Ilias D. Iliopoulos, Evangelia Papadimitriou, Efstathios Chronopoulos

**Affiliations:** 1 Laboratory of Molecular Pharmacology, Division for Orthopaedic Research, School of Health Sciences, University of Patras, 26504 Patras, Greece; epapad@upatras.gr; 2 First Department of Orthopaedic Surgery, “ATTIKON” University Hospital, School of Medicine, National and Kapodistrian University of Athens, Rimini 1, 12462 Athens, Greece; olgasavvidou@gmail.com (O.D.S.); dimitramelissaridi@gmail.com (D.M.); styliani.iliopoulou@hotmail.com (S.I.-K.); 3 Third Department of Orthopaedic Surgery, “KAT” General Hospital, School of Medicine, National and Kapodistrian University of Athens, Nikis 2, 14561 Athens, Greece; eliasvasiliadis@yahoo.gr; 4 Third Department of Orthopaedic Surgery, “KAT” General Hospital of Athens-NHS, Nikis 2, 14561 Athens, Greece; ortho.argiris@gmail.com; 5 Department of Orthopaedic Surgery, “Rion” University Hospital and Medical School, School of Health Sciences, University of Patras, 26504 Patras, Greece; iliopoulos.d.il@gmail.com; 6 Laboratory for Research of the Musculoskeletal System, School of Medicine, National and Kapodistrian University of Athens, 14561 Athens, Greece; stathi24@yahoo.gr

**Keywords:** neurofibromatosis type 1, scoliosis, bone metabolism defects, bone mineral density

## Abstract

Neurofibromatosis type 1 (NF1), which is the most common phacomatoses, is an autosomal dominant disorder characterized by clinical presentations in various tissues and organs, such as the skin, eyes and nervous and skeletal systems. The musculoskeletal implications of NF1 include a variety of deformities, including scoliosis, kyphoscoliosis, spondylolistheses, congenital bony bowing, pseudarthrosis and bone dysplasia. Scoliosis is the most common skeletal problem, affecting 10–30% of NF1 patients. Although the pathophysiology of spinal deformities has not been elucidated yet, defects in bone metabolism have been implicated in the progression of scoliotic curves. Measurements of Bone Mineral Density (BMD) in the lumbar spine by using dual energy absorptiometry (DXA) and quantitative computer tomography (QCT) have demonstrated a marked reduction in Z-score and osteoporosis. Additionally, serum bone metabolic markers, such as vitamin D, calcium, phosphorus, osteocalcin and alkaline phosphatase, have been found to be abnormal. Intraoperative and histological vertebral analysis confirmed that alterations of the trabecular microarchitecture are associated with inadequate bone turnover, indicating generalized bone metabolic defects. At the molecular level, loss of function of neurofibromin dysregulates Ras and Transforming Growth factor-β1 (TGF-β1) signaling and leads to altered osteoclastic proliferation, osteoblastic activity and collagen production. Correlation between clinical characteristics and molecular pathways may provide targets for novel therapeutic approaches in NF1.

## 1. Introduction

Neurofibromatosis type 1 (NF1), also termed von Recklinghausen disease, is referred to by the National Organization for Rare Disorders (NORD) as a rare autosomal dominant genetic disorder that affects 1 per 3000–6000 individuals worldwide [1,2,3]. NF1 is caused by loss-of-function mutations in the NF1 gene, which is located on the long arm of chromosome 17q11.2 and is composed by 60 exons spanning more than 350 kb of genomic DNA and encodes an intracellular protein called neurofibromin (Nf), which is responsible for the disease [4]. Nf is a cytoplasmic protein consisting of 2818 amino acids and expressed in many types of cells, including osteoblasts, osteoclasts, chondrocytes and neurons of the central and peripheral nervous system [5]. It is involved in the negative regulation of cellular proliferation, growth and differentiation through the inactivation of the Ras-GTPase protein and accumulation of cyclic adenosine monophosphate (cAMP) [6], being a significant tumor suppressor [7,8]. The regulatory activities of Nf in cellular functions are achieved via its implication in two major signaling pathways: (a) the Ras downstream signaling of Raf-MEK-ERK (Rapidly accelerated fibrosarcoma, Mitogen-activated protein Kinase and Extracellular signal Regulated Kinases, respectively) and (b) PI-3-K (phosphatidylinositol-3- phosphate kinase) pathways [6].

NF1 is an autosomal dominant disorder with 100% penetrance with a great variance in clinical presentation and relatively minor contribution of the nature of the NF1 mutation to disease expression. The diagnostic algorithm is based on the criteria of the USA National Institute of Health (NIH) [9] and/or the mutation analysis of the NF1 gene. Clinically, it is characterized by café-au-lait spots, intertriginous freckling, Lisch nodules, neurofibromas, optic pathway gliomas, distinctive bony lesions, malignant peripheral nerve sheath tumors, neurocognitive defects, epilepsy and cardiovascular abnormalities [10].

Individuals with NF1 are prone to developing a wide range of osseous and skeletal manifestations, such as macrocephaly, short stature, sphenoid wing dysplasia, scoliosis, congenital pseudarthrosis of the long bones [11,12], increased fracture risk [13], reduced bone mineral density (BMD) or osteoporosis [14,15,16]. Osteoporosis is found in 20–50% of NF1 patients, and it is associated with reduced serum 25-hydroxyvitamin D and increased serum concentrations of parathyroid hormone (PTH) and biochemical markers indicating bone turnover, such as osteocalcin or alkaline phosphatase (ALP) [17]. Bone specimens from NF1 patients demonstrate reduced trabecular volume, increased osteoid mass and elevated undifferentiated osteoblastic/osteoclastic cell count [18,19]. However, the pathophysiological mechanism that leads to bone mass reduction has not been elucidated yet. Nf gene deletion results in a pathological increase in intracellular Ras activity, induction of osteoclastic activity and inhibition of osteoblastic differentiation [18,19,20]. In vivo studies have suggested that dysregulation of transforming growth factor-β1 (TGF-β1) signaling correlates with progressive skeletal defects in Nf knockout mice, implicating TGF-β1 in the NF1 skeletal phenotypes [20].

Scoliosis is the most common musculoskeletal disorder of NF1 patients and might be accompanied by spinal dysplastic defects [21]. Many research studies have focused on BMD, as well as on biochemical and molecular indices, which were remarkably reduced in scoliotic patients revealing a link between spinal deformities and generalized metabolic osseous disease in NF1 [22]. Furthermore, novel bone anabolic therapies have been shown to promote osteogenic differentiation and to improve skeletal defects in NF1 [23]. Due to the limited number of literature reports on the association between scoliotic progression and bone metabolic impairment in NF1, the focus of our review study is to provide data on the following: (a) clinical presentation and associated demographic data, (b) characteristic biochemical bone metabolic alterations and (c) possible cellular and molecular signaling pathways that accompany scoliosis in NF1 patients.

## 2. Epidemiological Data and Clinical Characteristics

Despite the fact that spinal deformities in patients with NF1 were first reported by Gould in 1918 [24], very few epidemiological studies have examined the prevalence of scoliosis in this population. We must note that in the included studies, the diagnosis of NF1 and scoliosis was based on NIH and Scoliosis Research Society (SRS) criteria, respectively. However, the exact prevalence of scoliotic malformations in NF1 patients has not been clarified yet. In a cohort study of 438 children with NF1, Toro et al. [21] reported that the prevalence of scoliosis was 9.8%. Similar findings were reported by Alkbarnia et al. [25], while Lykissas [26] and Durrani et al. [24], who focused on the surgical restoration of the vertebral deformities, reported an incidence of 19% and 20%, respectively [26]. In a large epidemiological study of 3505 NF1 patients that were registered at the Japanese Ministry of Health between 2001 and 2014, which investigated the accompanied major complications requiring medical intervention, scoliotic manifestations were present in 10% of the affected population [27]. Interestingly, 55% of the patients with spinal deformities were prone to conservative or surgical forms of interventions [27].

Additionally, a retrospective analysis of 537 individuals using whole-body magnetic resonance imaging (MRI) with volumetric analysis detected an increased prevalence of spinal abnormalities in patients with NF1, as the incidence of scoliosis and neuroforaminal tumors were 46.9% and 39.6%, respectively [28]. In the same study scoliotic deformities in NF1 were linked to scalloping, meningoceles, neuroforaminal tumors and dural ectasia, demonstrating the importance of whole-body MRI in the evaluation of phenotype of spinal abnormalities in NF1 patients [28] as it provides a significant imaging biomarker not only for the assessment of tumor progression, but also for the study of treatment response. Indeed, it was reported that MRI was also used to examine bone marrow changes associated with osteoporosis in the axial and appendicular skeleton after the administration of imatinib in children and young adults with NF1 [29].

**Figure 1:**
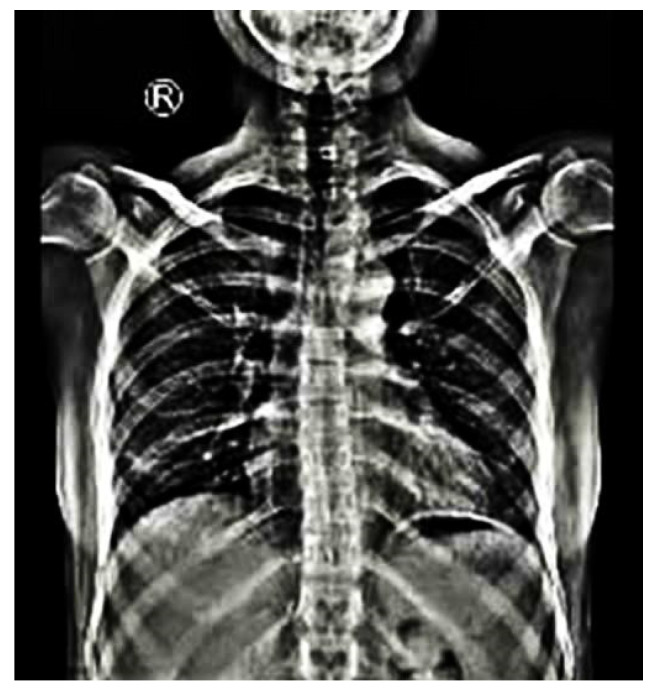
An anteroposterior view of total spine in standing position of a 30-year-old female patient with NF1 demonstrates a right thoracic curve of 12 degrees between 5th and 10th thoracic vertebras without signs of dystrophic malformations. (R: Right).

Predicated on the location of the apical vertebrae, scoliosis in NF1 can be classified in two main categories: non-dystrophic and dystrophic. The non-dystrophic scoliotic curves show radiological signs similar to adolescent idiopathic scoliosis (Figure 1) and can aggressively progress to the dystrophic phenotype [30]. Contrariwise, dystrophic malformations develop earlier than non-dystrophic, having a characteristic radiological appearance of sharp and angular curves with severe apical rotation that affect four to six vertebras [30]. The definition criteria of dystrophic scoliosis are presented in detail in Table 1.

In the study by Toro et al. [21], the prevalence of dystrophic scoliosis in the NF1 population was 39%, while in the study of Lykissas et al. [26] it was 63%. The above differences may be explained by the different diagnostic approaches and imaging modalities that were followed in each survey. Although the possibility of dystrophic deformities was equal between sexes [24,26], it was reported that in patients that were surgically treated, the male to female ratio was 4:1 [24]. Further analysis of the disclosed radiological dystrophic signs revealed that the most common characteristics were: (a) paravertebral neurofibromas in 22% of NF1 patients, (b) vertebral scalloping in 16% of the cases, and (c) short, segmented curve and anomalies of the dural sac, such as ectasia, syringomyelia and Chiari malformation [21,26]. Since a short segmental curve was also detected at an increased rate (~57%) in non-dystrophic scoliosis, it has been suggested that it is not a key dystrophic feature [21,26].

## 3. Bone Metabolism in NF1

As already mentioned, NF1 is associated with a remarkable reduction in BMD and increased osteopenia, osteoporosis and severe scoliosis [22,31,32,33]. In specific, Petramela et al. [31] reported an increased prevalence of osteopenia (44%) and osteoporosis (18%) in NF subjects compared to a normal control group. Similarly, Illes et al. [33], using dual-energy X-ray absorptiometry (DEXA), observed reduced Bone Mineral Density (BMD) in the lumbar spine of NF1 patients. Additionally, an inverse correlation was observed between the degree of scoliotic curves and BMD values, as in patients with an increased magnitude of spinal deformities, poor lumbar BMD was noted [33]. These results were in line with the data of Brunetti-Pierri et al. [22], which described the generalized reduction of spinal, femoral and trochanteric BMD, as well as whole-body bone mineral content (BMC) in NF1. Notably, the lumbar spine seems to be most severely affected [22]. Histological analysis of bone vertebral specimens that were received intraoperatively from NF1 patients with scoliotic deformities, revealed a marked reduction in bone trabeculae thickness and in viable osteocytes compared to healthy controls. Moreover, osteoblasts and bone lining cells were not well differentiated and were accompanied by a low number of active osteoblasts [22]. Cellular histomorphometry analyses also detected altered osteoblastic and osteoclastic populations in bone biopsies, indicating an increased turnover in NF1 patients that lead to heterogenic mineral and osteoid distribution and low calcium content [17,34]. These findings are confirmed in a more recent study that showed that trabecular bone score, bone mineralization and BMD are decreased in NF1 patients and are associated with severe spinal osteopenia and osteoporosis [35]. Similar results have been reported by several pediatric cohort studies [36,37,38,39]. Specifically, in NF1 children, non-dystrophic mild scoliotic curves have been correlated with low total body and lumbar Z-scores (≤−2) and low BMD. Interestingly, children with low Z-scores have a four-times greater risk of developing skeletal deformities when compared to children without spinal malformations [36]. These findings are consistent with the notion that low lumbar spinal trabecular BMD and Z-scores are predictors of scoliosis severity in NF1 children aged 6 to 9 years old [40]. The cohort study by Stevenson et al. [41], which investigated the contribution of low BMD and BMC of skeletal dysplasias in 84 children with NF1 compared to 293 healthy individuals, suggested that generalized osteopenia and/or osteoporosis are important predisposing factors of localized osseous defects. Contrariwise, Duman et al. found low femoral and lumbar BMD in pubertal children with NF1, but without being statistically correlated with skeletal abnormalities [39].

Regarding the status of bone metabolic biochemical and molecular markers, it has been reported that they are significantly affected in NF1 patients. Severe hypovitaminosis D, which is defined by serum levels lower than 20 ng/mL, is observed in more than 60% of patients with NF1 with scoliotic deformities [31]. Similarly, an increased incidence of osteomalacia associated with low serum concentrations of Vitamin D and increased bone turnover markers were also detected in NF1 individuals with scoliotic deformities [17,42]. Vitamin D insufficiency was detected in 75% of patients with NF1 in the study of Brunetti et al. [22]. Interestingly, children with NF1 showed higher levels of Vitamin D compared to adults [43], whereas in female pediatric patients, a negative correlation between serum levels of 25-OH vitamin D and lumbar Z-scores compared to males was noted [44]. Although oral administration of vitamin D resulted in normalization of 25OH-vitamin D serum levels [22,31], it was not associated with the restoration of lumbar BMD or whole-body BMC [22]. A possible explanation for this observation could be that NF1 neurofibromas were linked to the reduced expression of Vitamin D Receptor (VDR) [45]. VDR is a nuclear receptor which acts as transcriptional factor activated by 1,25(OH)_2_-D binding [46]. As VDR was immunohistochemically undetected in NF1-related tumors [45], we can hypothesize that the recovery of BMD and/or BMC in NF1 may be achieved, not only by the normalization of Vitamin D blood levels, but also by increasing the interaction between vitamin D and VDR [22,45]. Other factors that have been shown to affect bone homeostasis in NF1 are the elevated renal excretion of calcium, reduced levels of total and ionized plasma calcium and magnesium levels, and increased circulating levels of parathormone (PTH) that were associated with secondary hyperparathyroidism [17,31,39,44,47]. However, serum levels of inorganic phosphorus are not altered [17,39,47].

Assessment of bone turnover markers displayed increased levels of alkaline phosphatase (ALP) and osteocalcin [17,31,39,44,47]. Increased bone tartrate-resistant acid phosphatase (bone TRAP5b) serum and urine deoxypyridinoline cross-links were also observed and were associated with increased osteoclastic activity [48]. These findings were also confirmed by the study of Stevenson et al., which evaluated the urinary excretion of pyridinium crosslinks, such as pyridinoline (Pyd) and deoxypyridinoline (Dpd) in NF1 children with localized skeletal dysplasias including scoliosis [49], suggesting that the elevated ratio of Dpd/Pyd indicated a preferential increase in bone resorption rather than a generalized collagen breakdown [49]. Despite the fact that the referred biochemical bone markers have been correlated with an increased bone turnover and remodeling process [31], several studies did not reveal a significant statistical correlation between other bone markers, such as β c-terminal telopeptide, and densitometric results [35,44,47], suggesting the absence of accurate and predictive markers for NF1 and localized skeletal defects.

A rare entity, which is characterized by significant low levels of serum and increased concentrations of urine phosphate and leads to abnormal bone mineralization, is hypophosphataemic osteomalacia (HO) secondary to NF1. HO is a paraneoplastic syndrome that is accompanied by hypophosphataemia, hyperphosphaturia secondary to reduced proximal renal tubular phosphate reabsorption, and low or inappropriate normal levels of serum vitamin D. Moreover, serum concentrations of calcium and parathormone (PTH) were in normal levels, while calcium levels in urine were low [50]. NF1 patients with HO had low BMD, suffering from diffuse osteopenia [42,51] or osteoporosis [52,53,54] and bone demineralization [54,55] associated with several skeletal defects, such as scoliosis, kyphosis, bowing of long bones, pseudofractures, fractures and triradiate pelvis. The involvement of fibroblast growth factor 23 (FGF23) in the pathophysiology of NF1 bone defects with HO has also been proposed. FGF23, which is a phosphotropic hormone produced by bones [56], is mainly expressed in bony tissues, especially in osteoblasts/osteocytes, and exerts its action, after proteolytic activation, by binding to the FGF receptor-Klotho complex. Increased secretion of FGF23 from Nf-deficient osteocytes results in mineral defects and an osteomalacia-like bone phenotype [53] and has been associated with abnormal calcium-phosphorus metabolism and reduced bone formation and mineral apposition rate [57]. A possible explanation could be that the increased serum concentration of FGF-23 inhibited renal reabsorption of phosphorus and decreased the production of 1,25-dihydroxy- vitamin D leading to increased phosphate wasting and lower levels of phosphorus in the serum [57].

## 4. Molecular Basis for Skeletal Deformities in NF1

**Figure 2:**
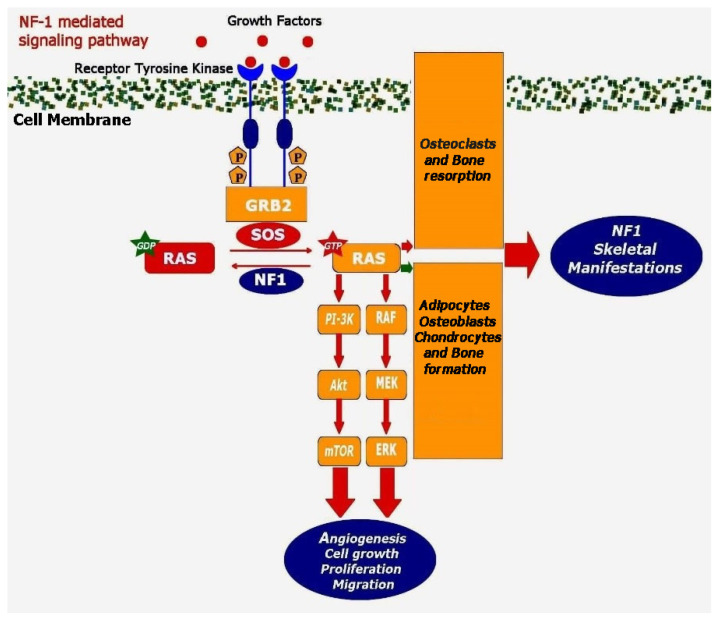
Nf1 is a GAP-like protein located in cytoplasm and negatively regulates the activation of the RAS signaling pathway by converting RAS-GTP to RAS-GDP. The RAS-GTP activates both PI-3-K and classical MAPK pathways, resulting in the regulation of several cellular functions, such as angiogenesis, cell growth, proliferation and migration. Inhibition of Nf induces RAS activity and the signaling cascade of the MEK/ERK and Akt/mTOR pathway. Nf is also a key regulator of bone development and repair. Inhibition of Nf and the induction of RAS signaling pathway augments the expression of osteoclastic cell lines (red arrows) and declines the osteogenic differentiation (green arrows), resulting in skeletal defects. Akt, Protein kinase B; ERK, extracellular signal-regulated kinase; GDP, guanidine diphosphate; GRB2, growth factor receptor-bound protein 2; GTP, guanidine triphosphate; MAPK, mitogen-activated protein kinase; MEK, MAPK/extracellular-signal-regulated kinase; mTOR, Mechanistic Target of Rapamycin Kinase; NF1, Neurofibromatosis type 1; Raf, serine/threonine-protein kinase; SOS, son of sevenless.

Given the paucity of human clinical studies on skeletal development and bone remodeling, insights into the exact implication of Nf in osteoblastic and osteoclastic activity come from in vitro and in vivo experimental studies. It has been well established that Nf directly affects the Ras downstream signaling of Raf-MEK-ERK and PI-3-K pathways (Figure 2), which interact with pathways of high importance for spinal development and bone repair [58].

Nf1 via regulation of RAS signaling modulates both anabolic and catabolic pathways of bone homeostasis and affects spinal formation and remodeling process [58]. Activation of RAS subsequently stimulates RAF protein which in turn activates MEK protein via phosphorylation. The activated MEK then phosphorylates and activates MAPK and, ultimately, this signaling cascade results in cellular growth, migration and proliferation. Another pathway that is negatively regulated by Nf1 is the RAS-mTOR signaling pathway, which also promotes cell growth and proliferation, and Nf-deficient cells experience continuous activation of the RAS-MAPK and RAS-mTOR pathways. Upregulation of RAS-MAPK signaling in osteoclastic cell lines triggers growth and survival, leading to bony tissue and matrix degradation defects. Inactivation of Nf1 and the associated dysregulation of Ras signaling also impairs osteoblastic differentiation from mesenchymal stem progenitor cells (MSPC) (Figure 2). In vitro studies displayed that MSPC collected from heterozygous knockout mice (Nf1^+/−^) have impaired osteoblast differentiation, as determined by ALP staining and CFU-F replating assays. This impaired osteogenic differentiation is in line with the decreased mRNA levels of osteocalcin and osteonectin, while there are no signs of chondrocyte differentiation. Interestingly, expression of the NF1 GTPase activating-related domain (NF1 GAP-related domain) increased osteoblast formation and differentiation in Nf1^+/−^ MSPC [19]. These findings were in line with the observations of Yu et al. [59] that Nf1^+/−^ osteoprogenitors exhibit premature apoptosis and reduced induction of osteoblastic differentiation [59]. Analysis of Ras activity levels revealed that Nf1^+/−^ osteoprogenitors express increased basal and PDGF-stimulated Ras-GTP levels compared to Nf1^+/+^ osteoprogenitors, suggesting that Nf regulation of Ras is required for the induction of osteoblast differentiation [59]. A recent study of Ma et al. [60] noted an upregulation of inorganic pyrophosphate (PPi) pathway-related genes in Nf1^−/−^ osteoprogenitor cells and in NF1 human Schwann cells, such as Enpp1 (ectophosphatase generating PPi), Ank (channel transporting PPi in the extracellular matrix) and osteopontin. Concurrent in vivo studies of Nf conditional knockout models showed that vertebral processes adjacent to large paraspinal plexiform neurofibromas are completely unmineralized [60]. Similarly, altered calcium-phosphorus metabolism is accompanied by a reduced number of osteoblasts and disorganized osteocyte dendrites conducing a severe reduction in mineral apposition, mineralized surface and bone formation rate in the trabecular bones of these mice [53]. The importance of the Ras-MAPK pathway is supported by the observation that administration of selumetinib, which is a selective MEK inhibitor, improved BMD in an NF1 patient, providing evidence that MEK inhibitors may be helpful in diseases caused by bone mineralization deficiencies [60]. It should be noted, however, that although Nf1^+/−^ mice have a decreased periosteal and endocortical bone formation and significantly reduced bone formation rate, the overall bone mass and geometry is not affected, indicating that unknown compensatory pathways may control Ras signaling to maintain normal bone mass and function in vivo, especially in the heterozygous forms of NF1 [59].

In NF1, the PI3K-AKT-mTOR pathway also seems to be involved in the deranged osteogenic differentiation of BMSC [61,62]. The down-regulated expression of Nf in human BMSC results in enhanced mTORC1 activity and a remarkable reduction in osteoblastic differentiation markers, such as osterix, runx2 (RUNX Family Transcription Factor 2) ALP and OCN, while overexpression of Nf1 had the opposite outcome [61]. Some studies suggest that the osteogenic differentiation is strongly dependent on autophagy [62], with mTORC1 playing a regulatory role in this process [63,64]. Overexpression of Nf in BMSC inhibits mTORC1 signaling and thus enhances autophagy and results in new bone formation [64]. In the same line, inhibition of Nf1 in BMSC enhances mTORC1 signaling and decreases the expression of autophagy markers, such as Beclin-1 and LC3B-II, as well as bone differentiation markers, such as osterix, runt-related transcription factor 2 and ALP [62]. Moreover, in the Nf1-siRNA group the activity of the PI3K/AKT/mTOR pathway was significantly upregulated, whereas administration of the autophagy activator RAPA reserved the knockdown effects of Nf1-siRNA on the autophagy and osteogenic differentiation of BMSCs and led to elevated ALP activity and calcium deposition [62].

Both laboratory and experimental results suggest that bone catabolic pathways are activated in NF1. Osteoclasts isolated from Nf1^+/−^ mice, cultured in the presence of recombinant receptor activator of nuclear factor-κΒ ligand (RANKL) and macrophage colony-stimulating factor (M-CSF), display enhanced Akt phosphorylation, survival, proliferation, migration and adhesion in vitro. These observations are in line with the severe osseous defects in ovariectomized Nf1 knockout mice, as well as data from in vitro differentiated osteoclasts collected from NF1 patients that have an activated Ras/PI3K pathway and increased ostolytic activity [18]. Taking into consideration that the combination of Ras-MAPK activity and RANKL production is critical for the regulation of osteoclastic functions [65,66,67,68], they may trigger the bone catabolic processes in NF1 [69].

In vivo models of experimentally induced scoliosis noted an increased expression of angiogenesis-regulating factors, such as metalloproteinase-1 (MMP1) and -12 (MMP12), vascular endothelial growth factor A (VEGFA) and pleiotrophin, supporting the notion of a close connection between defective angiogenesis and scoliosis progression [70,71,72,73]. The Nf1 gene regulates Ras-related signaling pathways that are involved in angiogenesis regulation (Figure 2), while altered vascularization was observed in in vitro and in vivo studies investigating NF1 pathophysiology [74,75]. Nf1 deficiency in mice is accompanied by the enhanced expression of fibroblast growth factor 2 (FGF-2), platelet-derived growth factor (PDGF) and midkine (MK) [74]. Furthermore, endothelial cells from Nf1^+/−^ mice demonstrate increased migration and proliferation in response to mitogens and to FGF2 in vitro and in vivo, respectively, resulting in increased neovascularization in both the retina and cornea [75]. Immunohistochemical methods displayed increased generalized vascular endothelial thickening around NF1-related congenital tibial pseudarthrotic tissue [76]. Impaired angiogenesis was also observed in abnormal periosteum in an NF1 patient, suggesting a link with decreased osteogenic capabilities in NF1 [77]. Furthermore, an immature and defective vascular network was associated with impaired porous formation and non-union after a Masquelet reconstruction technique for bone defects [78]. Therefore, flawed neovascularization may be correlated with delayed bone development and/or repair, potentially contributing to skeletal and spinal deformities in NF1.

**Figure 3:**
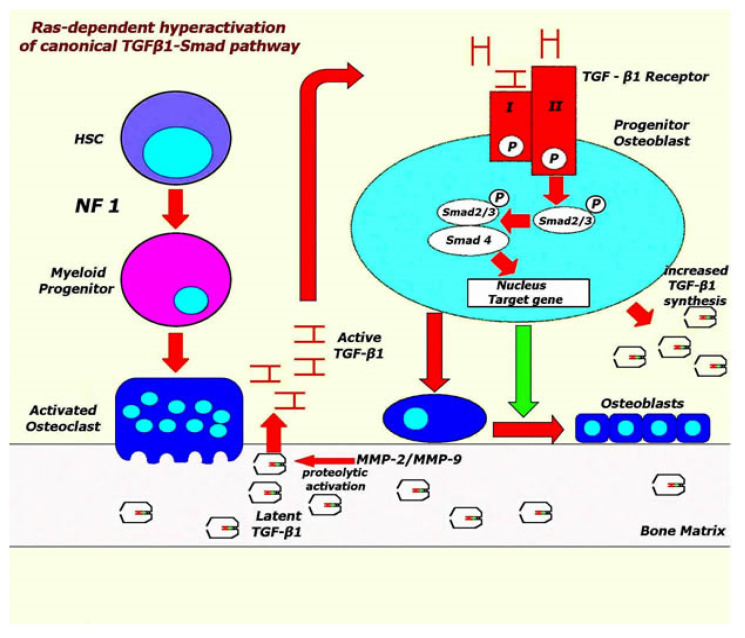
Nf is a negative regulator of TGF-β1 signaling pathway. Inhibition of Nf activity was associated with p21-Ras-dependent hyperactivation of the canonical TGFβ1-Smad pathway that resulted in increased expression of TGF-β1 potentiating osteoclastic activation (red arrows) and inhibiting osteoblastic differentiation (green arrow) via MMP-2/MMP-9 proteolytic activation of the latent TGF-β1 (prodomain structure of TGF-β1). The model has been proposed by Rhodes et al. [20] describing the NF1-associated skeletal deformities mediated by the pathological cycle of increased TGFβ1-Smad signaling. HSC, Hematopoietic stem cells; MMP-2, -9, Matrix metalloproteinases 2, 9; NF1, Neurofibromatosis type 1; TGF-β1, Transforming growth factor beta 1.

Oligonucleotide-based array analysis to examine the expression pattern of blood cell genes has revealed that the expression of several genes implicated in calcified tissue remodeling and bone development was down-regulated, while TGF-β1 was increased in NF1 patients [79]. Similarly, in a mouse model of NF1, serum levels of total TGF-β1 in Nf1 conditional knockout mice have been found to be significantly increased compared to wild type mice and have been associated with multiple skeletal abnormalities, such as osteoporosis and impaired fracture healing. Hypersecretion of TGF-β1 has led to an increased activation of the canonical Smad pathway and to pathological osteoblastic and osteoclastic differentiation, contributing to increased bone resorption. In the same study, increased levels of active MMP2 and MMP9 in both the Nf1 haploinsufficient myeloid cells and the serum of a human NF1 patient were also noted. These functions were reserved by the re-expression of full-length Nf1 in primary Nf1-deficient osteoblast progenitors that resulted in reduced Smad phosphorylation (Figure 3). Finally, treatment with TGF-β receptor 1 (TβRI) kinase inhibitor rescued defects of BMD and enhanced tibial fracture healing in Nf1 conditional knockout mice [20]. Taking into account the data from several genetic and experimental studies that unveiled the pathophysiological contribution of TGF-β1 signaling in syndromes, such as Camurati-Engelmann [80,81], Loeys-Dietz [82,83], Shprintzen-Goldberg [82,84,85] or Marfan disease [86,87], and their clinical presentations with severe skeletal malformations including altered remodeling, osteoporosis and dystrophic scoliotic deformities, the link between scoliosis or other bone defects in NF1 and induction of TGFβ1-Smad axis is an interesting hypothesis.

## 5. Conclusions

Collectively, bone metabolic impairment, consistent with the impairment of osteoblastic expression and osteoclastic activity and the associated progressive decrease in bone mass, contribute to the severity of NF1 deformities in the anatomic locations where increased mechanical forces are applied, such as the spine or tibia. Although genetic or epigenetic factors may affect the severity of skeletal dysplasias [58], the correlation of dystrophic scoliotic curves, dysplastic vertebral elements and pseudarthrosis with deficits in metabolic phenotype and in bone repair process, could be contributors to the progression of deformities, providing targets at a molecular level for novel therapeutic approaches to improve the long-term outcome of surgical and/or conservative interventions for the management of scoliotic malformations in NF1.

## Figures and Tables

**Table 1:** Criteria for the definition of dystrophic scoliosis modified by Duranni [24] and Lykissas et al. [26]. Dystrophic scoliosis is diagnosed when three or more of the following criteria are fulfilled.

Scoliotic Vertebral Dystrophic Alterations
Vertebral scalloping (depth of scalloping more than 3 mm or 4 mm in the thoracic and lumbar spine, respectively) Rib penciling (rib width lower than the narrowest portion of the second rib) Spindling of the transverse processes (loss of 50% from the height of the transverse process) Vertebral rotation of grade 3 or more (according to Moe-Nash method) Focal, short-segmented curve (in 6 or less vertebrae) Dural ectasia Paraspinal tumors and/or plexiform neurofibromas close to scoliotic curves Vertebral wedging (in sagittal or coronal plane) Intervertebral foraminal widening Widened interpediculate distances Dysplastic pedicles
